# Pre-, Per- and post-cooling strategies used by competitive tennis players in hot dry and hot humid conditions

**DOI:** 10.3389/fspor.2024.1427066

**Published:** 2024-09-18

**Authors:** Nicolas Robin, Miguel Crespo, Toru Ishihara, Robbin Carien, Cyril Brechbuhl, Olivier Hue, Laurent Dominique

**Affiliations:** ^1^ Laboratory ACTES (EA 3596), Sport Sciences Faculty, University of Antilles, Pointe-à-Pitre, France; ^2^ International Tennis Federation, London, United Kingdom; ^3^ Graduate School of Human Development and Environment, Kobe University, Kobe, Japan; ^4^ Fédération Française de Tennis, Paris, France; ^5^ Laboratory IRISSE (EA 4070), Sport Sciences Faculty, University of La Reunion, Le Tampon, France

**Keywords:** cooling strategy, tennis, hot, environment, hygrometry, dry, humid, heat

## Abstract

**Purpose:**

This research investigated the pre-, per- and post cooling strategies used by competitive tennis players from various levels of play who occasionally train and compete in hot (>28°C) and humid (>60% rH), and dry (<60% rH) environments.

**Methods:**

129 male tennis players (M_age_ = 24.9) competing at regional (*N* = 54), national (*N* = 30) and international (*N* = 45) levels, completed an online questionnaire regarding their use (i.e., timing, type, justification and effectiveness) of pre- (i.e., before practice), per- (i.e., during exercise) and post-cooling strategies when playing tennis in hot dry (HD) and hot humid (HH) conditions. Individual follow-up interviews were also carried on 3 participants to gain an in-depth understanding of the player's experience.

**Results:**

Competitive tennis players used both internal and external cooling strategies to combat the negative effects of HD and HH conditions, but considered the HH to be more stressful than HD and experienced more heat-related illness in HH environments. International players used cold packs and cold towel more frequently than the regional and national players in hot environments, and used cold water immersion and cold vest more frequently than the latter in HH. Differences in strategy use were mostly observed during per-cooling where regional and national players more frequently used cold drinks than international players who more frequently used cold packs in HD and cold towel in HH conditions. Moreover the latter more frequently used cold towel, cold packs and cold water immersion as post-cooling strategies than regional players.

**Conclusion:**

When playing tennis in the heat, it is strongly recommended to employ cooling strategies to maintain health, limit declines in performance, and promote recovery. We also recommend improving education regarding the appropriate use and effectiveness of cooling strategies, and increasing their availability in tournaments.

## 1 Introduction

Tennis is a racket sport that involves hitting a ball and requires the development and use of technical, tactical, mental and physiological skills (1). It is a game that requires short and high-intensity repetitive sprints using explosive strength from the legs combined with muscular strength at the level of the upper limbs and shoulders (2). Added to this is the need for a high degree of attentional processes, visuomotor timing and visual tracking. The tennis player has to perform all these activities for around an hour and a half until more than five hours (3). Regarding physiological demands, tennis is considered a predominantly anaerobic sport that requires a high level of aerobic quality to facilitate the player's recovery between points and matches (4) during tournaments.

About 30% of the International Tennis Federation tournaments take place in hot (>28°C) dry or humid conditions (5). The thermal stress of a hot environment imposes additional physiological constraints on the cardiovascular system (6). The latter, during a long-lasting effort such as a tennis match or a training session, must evacuate the heat produced by the muscles (metabolic heat), and possibly the heat from the environment, to maintain a constant body temperature (3). Thermoregulation processes (i.e., conduction, convection, radiation and evaporation) allow tennis players to maintain their internal temperature through heat exchange between the skin and the environment (1, 4, 7).

In neutral conditions (neither too hot: <25°C nor too humid: <50% rH), the physiological mechanisms of thermoregulation generally allow to compensate for the production of body heat, and the core temperature will stabilize around 38.5°C (8]). However, the combination of exercise during tennis practice and the thermal stress induced by a hot environment (>25°C) will lead to an increase in body temperature (3). This increase has been shown to be mainly linked to cardiovascular, neuromuscular, metabolic alterations and sweat evaporation capacities (9), the convection and conduction mechanisms being limited (10).

It is very important to emphasize that in hot conditions, the degree of relative humidity can have a considerable impact on thermoregulation (11). Indeed, in a hot environment with low relative humidity (<60% rH), sweat produced by the skin will easily evaporate and heat will be transferred to the environment at high rates (12). However, in a hot and humid (>60% rH) environment, the sweat evaporation capacity will be reduced (13) and will be lower that in dry and hot conditions (14) that can facilitate a further increase in core temperature which can reach 39.5°C (3) because sweat evaporation is the main mechanism of removal of accumulated heat (7).

Studies have concluded that in hot conditions, decreases in heat elimination processes in the body and also in the brain can induce early mental fatigue (9) and can negatively impact cognitive (15), attentional (1) or motor (16) performances. To get an adequacy between the heat gain and the dissipation of the heat during the practice of the tennis in hot condition, athletes may be tempted to reduce metabolic heat production by decreasing exercise intensity (3). In addition, heat can negatively influence psychological factors such as thermal comfort, perceived exertion or thermal sensation, and the combination of warm ambient temperature and high levels of relative humidity will be worse (9). Finally, when exercising in hot conditions, tennis players can suffer from heat-related illnesses such as cramp, faintness, injury or heatstroke (17).

To maintain their health and limit decline in performance, players may utilize heat mitigation strategies such as fluid ingestion to limit dehydration (18) or acclimation (6), which generally involves arriving at the competition venue several days before the event to benefit from an optimal physiological adaptation and better withstand the heat (19). However, acclimation can be too costly for the players and also cause time constraints, which is why athletes resort more often to cooling strategies (20). Cooling strategies are generally categorized as external (ice towels, cooling vest, cooling garments, neck cooling, cold pack, menthol application, water spray, cool showers or cold water immersion) or internal (cold drink, ice slushy or ice slurry ingestions with or without menthol) that can reduce core and/or skin temperatures, perceived exertion and can improve performances and thermal comfort when exercising in the heat (21). In addition, cooling strategies can be used at different timings: before (pre-cooling), during practice or competition (per-cooling) and between matches or after exercises to facilitate recovery (post-cooling), and these strategies can be of different types.

Indeed, in a recent study that evaluated the use of cooling strategy in endurance athletes in hot conditions, Racinais et al. (22) revealed that most participants adopted pre- (mainly ice-slurry ingestion, neck collar, cold towel or ice west) and per- (head/face dousing and cold water ingestion) cooling strategies, primarily based on their personal experiences. In addition, when comparing the type of per-cooling strategies used in hot humid or dry conditions by elite triathlon or cycling athletes, Bayne et al. (11) found that regardless of relative humidity level, cold-water pouring was the most used strategy, mainly during pit stop. Moreover, the authors found that athletes’ preferred strategy in hot and dry (HD) condition was cold-water ingestion, whereas cold water pouring and cold-water ingestion were preferred in hot and humid (HH) environments. Finally, Bongers et al. (23) evoked that water dousing was particularly effective in hot and dry environments, but has limited cooling capacity in HH conditions. Therefore, different internal and external cooling strategies appear to be more and less effective in dry vs. humid conditions.

Tennis matches provide time to implement cooling strategies before playing and during exercise due to the break after the initial short warm-up and the breaks when switching sides after odd games and between sets (24). Most studies testing the effect of different cooling strategies when playing tennis in the heat involved simulated matches performed in a laboratory climate chamber (25–28). One of the few studies conducted under HD outdoor ecological conditions (33.6°C; 49% rH), showed that ice slurry ingestion and ice vest were the most effective cooling strategies to mitigate the development of heat strain in eight tennis players (24). However, the authors also mentioned that the ingestion of a large amount of ice slurry could cause gastric discomfort or bloating.

In addition to the need for a confirmation with a larger sample, the results of Naito's et al. study only looked at tennis matches played in a HD environment but did not investigate the HH conditions or if athletes who play tennis in hot conditions actually use cooling strategies whatever their competitive level. This study aimed to close these research gaps by evaluating the type, justification and perceived effectiveness of the pre, per- and post-cooling strategies employed by regional, national and international level tennis players who competed in HD as well as in HH environments.

## 2 Method

### 2.1 Participants

**Figure 1:**
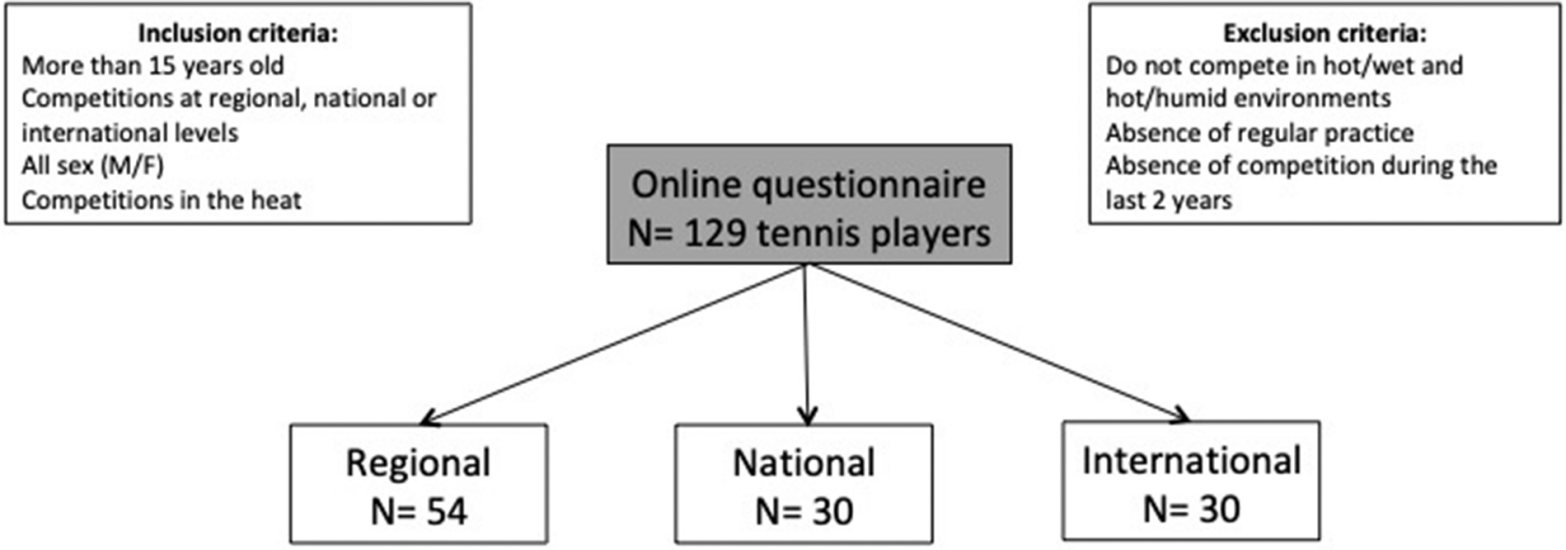
Flow chart.

One hundred and twenty-nine male tennis players (M_age_ = 25.9 ± 10.1 years; age range 18–52) competing at regional, national or international levels, volunteered to participate in the study (Table 1). Tennis players were from France (*N* = 77), United-States of America (*N* = 25), Japan (*N* = 10), Switzerland (*N* = 6), Indonesia (*N* = 3), Croatia (*N* = 2), New Zealand (*N* = 2), Belgium (*N* = 1), Peru (*N* = 1), Poland (*N* = 1), Turkey (*N* = 1), Moldova (*N* = 1). Those whose primary residence was located in hot environments were excluded from the study due to acclimatization effects (9). The players were informed that their participation was conditional on having trained and competed in both hot and dry (HD; >28°C; <60% rH) and hot and humid (HH; >28°C; >60% rH) conditions. They were divided into regional (*N* = 54), national (*N* = 30) and international (*N* = 45) categories according to their competitive level (Figure 1). This study was approved by the local ethics university committee and was carried in accordance with the declaration of Helsinki.

**Table 1:** Participant characteristics .

Level	Number of participants	Age (years)	Experience (years)	Hours training per week	Number of matches played in competition per year
All	129	24.9 ± 10.9	16.1 ± 7.7	11.6 ± 9.6	43.9 ± 24.5
Regional	54	28.3 ± 12.1	16.3 ± 8.2	5 ± 3.8	26.5 ± 15.6
National	30	24.4 ± 8.9	17.3 ± 6.9	9.8 ± 6.7	39.8 ± 20.4
International	45	21.4 ± 4.9	15.1 ± 6.5	22.1 ± 6.8	67.3 ± 15.2

### 2.2 Material and procedure

#### 2.2.1 Questionnaire

All the participants received a link by email, WhatsApp®, Twitter®, Facebook® or Instagram® allowing them to complete an online questionnaire. It was made clear to them that they must have played in both HD and HH conditions. After answering demographic questions (i.e., gender, age, country, place of life, tennis level, years of practice, symptoms related to heat-related illness in HD and HH conditions). The participants also had to indicate the number, location (or tournament name) and the date of the practice (training or competition) performed in HD and HH environments. Then, they add to answer questions concerning their use of pre-, per and post-cooling strategies in HD conditions. These questions concerned the timing (i.e., “*When was (were) the strategy (ies) used?*”), the type (i.e., “*What was (were) the cooling strategy (ies) employed?*”), the justification [i.e., “*Why did you use such cooling strategy (ies) at this time?*”] and the effectiveness of the strategy by using a Lickert-type scale from 1 “*not effective for minimising performance impairments and suffering heat-related illnesses*” to 5 “*Effective for minimizing both performance impairments and suffering from any heat-related illnesses*” [see (11) for similar procedure]. Then, the participant had to answer identical questions concerning the cooling strategies they employed in HH conditions.

#### 2.2.2 Interview

To support the answers to the questionnaire, 3 athletes (1 from each level category: regional, national and international) also completed individual online-recorded interview to a better understanding of the player's experiences concerning the use of cooling strategies [see (11) for similar procedure; and Supplementary Material file for the details and results].

### 2.3 Data analysis

For the questionnaire, the answers of the players concerning the timing, type, justification or perceived effectiveness were expressed as percentages values [see (11) for a similar procedure]. The recorded interviews were anonymized, transcribed and analysed according to the procedure of Braun and Clarke (29). To test the differences in symptoms and the use of cooling strategies by environments and players’ levels, *χ*^2^ tests were performed. Subsequently, stratified analyses were performed to assess differences between environments within each player level and vice versa. Multiple comparison adjustments were applied using the Bonferroni method. The significance level was set at *α* = 0.05.

## 3 Results

The detailed results of *χ*^2^ tests are summarized in the Supplementary results.

### 3.1 Condition

More than 98% of the regional, national and international players felt a difference when playing in HD compared to HH conditions, with the HH environment considered to be the most stressful in 74%, 87% and 84% of them, respectively (*χ*^2^ = 9.2, *p* = 0.002). The chi-square test showed no credible differences among players’ levels (*χ*^2^ = 2.6, *p* = 0.27).

### 3.2 Symptom

As illustrated on Table 2, 70%, 47% and 52% of the regional, national, and international players, respectively, experienced any symptoms related to heat-related illness in HD conditions. They were 80%, 77% and 69%, respectively, in HH environments. The chi-square test revealed a higher prevalence of symptoms in HH compared to HD conditions (*χ*^2^ = 9.3, *p* = 0.002), while we found no credible evidence of differences across players’ levels (*χ*^2^ = 5.5, *p* = 0.06). Stratified analyses showed that the differences between environments are significant for national players (*χ*^2^ = 7.2, *p* = 0.007), while we found no credible evidence of differences for regional players (*χ*^2^ = 1.2, *p* = 0.27). National (*χ*^2^ = 7.5, *p* = 0.006) and international (*χ*^2^ = 4.1, *p* = 0.04) players perceived more cramps in HH than HD conditions and international players indicated more heat exhaustion in HH than in HD (*χ*^2^ = 4.5, *p* = 0.03).

**Table 2:** Symptoms related to heat-related illnesses in hot and dry (HD) and hot and humid (HH) conditions depending on the level of the players.

Level	Percentage of players who experienced any symptoms related to heat-related illness in hot (>28°C) and dry (<60% rH) conditions Types of symptoms experienced:	Percentage of players who experienced any symptoms related to heat-related illness in hot (>28°C) and humid (>60% rH) conditions Types of symptoms experienced:
Regional (*N* = 54)	70.3%	79.7%
	Muscle cramps: 37.0% (*N* = 20)	Muscle cramps: 38.9% (*N* = 21)
	Heat exhaustion: 40.7% (*N* = 22)	Heat exhaustion: 57.4% (*N* = 31)
	Heat-related injuries: 1.8% (*N* = 1)	Heat-related injuries: 1.8% (*N* = 1)
	Heat stroke: 20.4% (*N* = 11)	Heat stroke: 20.4% (*N* = 11)
National (*N* = 30)	46.7%	76.7%
	Muscle cramps: 16.7% (*N* = 5)	Muscle cramps: 30% (*N *= 15)
	Heat exhaustion: 30% (*N* = 9)	Heat exhaustion: 46.7 (*N* = 14)
	Heat-related injuries: 0% (*N* = 0)	Heat-related injuries: 0% (*N* = 0)
	Heat stroke: 3.3% (*N* = 1)	Heat stroke: 13.3% (*N* = 4)
International (*N* = 45)	51.1%	68.9%
	Muscle cramps: 22.2% (*N* = 10)	Muscle cramps: 42.2% (*N* = 19)
	Heat exhaustion: 33.3% (*N* = 15)	Heat exhaustion: 55.5% (*N* = 25)
	Heat-related injuries: 2.2% (*N* = 1)	Heat-related injuries: 2.2% (*N* = 1)
	Heat stroke: 13.3% (*N* = 6)	Heat stroke: 11.1% (*N* = 5)

### 3.3 Type of cooling strategies

**Figure 2:**
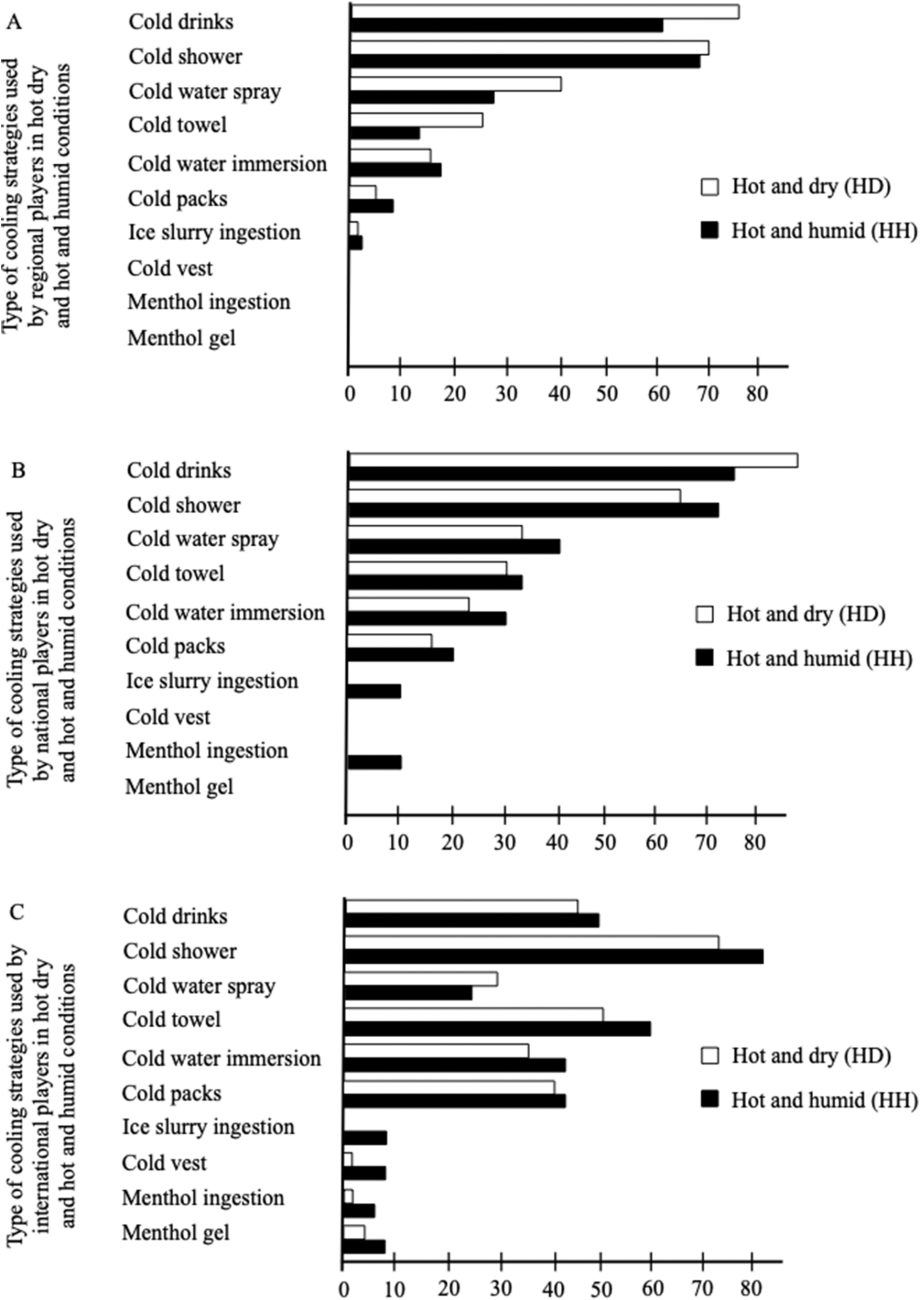
Type of cooling strategies employed by regional **(A)**, national **(B)** and international **(C)** players in hot and dry (white squares) and hot and humid (black squares) conditions.

Regional players declared using as cooling strategy, in HD and HH conditions, respectively: cold drinks (76%, 59%,) cold shower (68%, 67%), cold water spray (41%, 28%), cold towel (26%, 13%), cold water immersion (17%, 18%), cold packs (5%, 9%), ice slurry ingestion (2%, 4%) but not cold vest, neither menthol ingestion nor menthol gel (0%) in both conditions (Figure 2A).

National players reported using, in HD and HH conditions: cold drinks (87%, 73%), cold shower (63%, 70%), cold water spray (34%, 40%), cold towel (30%, 33%), cold water immersion (23%, 30%), cold packs (17%, 20%), menthol ingestion (7%, 7%), ice slurry ingestion (0%, 10%,) menthol gel (0%, 10%), but not cold vest (0%) in both conditions (Figure 2B).

International players used, in HD and HH environment, respectively: cold shower (71%, 80%), cold towel (49%, 58%), cold drinks (44%, 49%), cold packs (40%, 42%), cold water immersion (36%, 42%), cold water spray (29%, 24%), menthol gel (4%, 9%), cold vest (2%, 9%), menthol ingestion (2%, 7%), ice slurry ingestion (0%, 9%) as illustrated in Figure 2C.

Ice slurry ingestion was more frequent in HH compared to HD conditions (*χ*^2^ = 6.7, *p* = 0.01), while we found no credible evidence of differences between environments for other strategies (*χ*^2^ < 2.9, *p* > 0.08). Differences among players’ levels were detected for the use of cold drinks (*χ*^2^ = 18.8, *p* < 0.001), cold towels (*χ*^2^ = 25.3, *p* < 0.001), cold water immersion (*χ*^2^ = 11.3, *p* = 0.004), cold packs (*χ*^2^ = 33.3, *p* < 0.001), menthol ingestion (*χ*^2^ = 6.5, *p* = 0.04), menthol gel (*χ*^2^ = 7.0, *p* = 0.03), and cold vests (*χ*^2^ = 9.5, *p* = 0.009), while we found no credible evidence of differences among players’ levels for cold showers (*χ*^2^ = 1.9, *p* = 0.38) and cold water spray (*χ*^2^ = 2.0, *p* = 0.36).

The use of cold drinks was more frequent in regional and national compared to international players (*χ*^2^ = 8.8, adjusted *p* = 0.009; *χ*^2^ = 16.7, adjusted *p* < 0.001, respectively). Stratified analyses showed that these differences were observed only in the HD (*χ*^2^ = 17.7, *p* < 0.001) but not in the HH environment (*χ*^2^ = 4.4, *p* = 0.11). On the other hand, the use of cold towels and cold packs was more frequent in international compared to regional (*χ*^2^ = 24.8, adjusted *p* < 0.001; *χ*^2^ = 31.8, adjusted *p* < 0.001, respectively) and national (*χ*^2^ = 6.8, adjusted *p* = 0.03; *χ*^2^ = 8.6, adjusted *p* = 0.01, respectively) players. Stratified analyses showed that these differences were observed in both HD (*χ*^2^ = 6.1, *p* = 0.047; *χ*^2^ = 18.4, *p* < 0.001, respectively) and HH (*χ*^2^ = 22.2, *p* < 0.001; *χ*^2^ = 15.2, *p* < 0.001, respectively) environments.

The use of cold water immersion, menthol gel and cold vests was more frequent in international compared to regional players (*χ*^2^ = 11.2, adjusted *p* = 0.002; *χ*^2^ = 7.4, adjusted *p* = 0.02; *χ*^2^ = 6.2, adjusted *p* = 0.03, respectively). Stratified analyses showed that these differences in cold water immersion and cold vests were observed only in the HH (*χ*^2^ = 6.6, *p* = 0.04; *χ*^2^ = 7.7, *p* = 0.02, respectively) but not in the HD (*χ*^2^ = 4.7, *p* = 0.09; *χ*^2^ = 1.9, *p* = 0.39, respectively) environments. We found no credible evidence of differences in menthol gel in stratified analyses (HD: *χ*^2^ = 3.8, *p* = 0.15; HH: *χ*^2^ = 5.4, *p* = 0.07).

The use of menthol gel was more frequent in national compared to regional players (*χ*^2^ = 7.4, adjusted *p* = 0.02), while stratified analyses failed to detect a credible difference in the HD (*χ*^2^ = 3.8, *p* = 0.15) and HH (*χ*^2^ = 5.4, *p* = 0.06) environments.

### 3.4 Timing

There were seven strategies used in HD and HH conditions, by the regional players were respectively:

– Pre-cooling: cold drinks (33%, 33%), cold shower (24%, 24%), cold water spray (7%, 7%), cold water immersion (5%, 11%), cold towel (2%, 2%).
– Per-cooling: cold drinks (74%, 52%), cold water spray (41%, 38%), cold towel (26%, 13%), cold packs (4%, 7%), ice slurry ingestion (0%, 2%).
– Post-cooling: cold shower (65%, 57%), cold drinks (48%, 37%), cold water spray (17%, 9%), cold water immersion (15%, 17%), cold packs (2%, 5%), ice slurry ingestion (2%, 4%), cold towel (0%, 2%).

There were seven strategies employed in HD and nine in HH conditions, by the national players were respectively:

– Pre-cooling: cold drinks (30%, 30%), cold shower (13%, 23%), cold water spray (7%, 3%), cold water immersion (3%, 7%), menthol ingestion (3%, 0%), ice slurry ingestion (0%, 3%).
– Per-cooling: cold drinks (70%, 66%), cold water spray (36%, 30%), cold towel (30%, 27%), cold packs (10%, 17%), ice slurry ingestion (0%, 7%), menthol ingestion (3%, 3%), menthol gel (0%, 10%).
– Post-cooling: cold shower (60%, 63%), cold drinks (60%, 37%), cold water immersion (23%, 30%), cold water spray (17%, 10%), cold packs (13%, 7%), cold towel (3%, 10%), menthol gel (0%, 3%).

There were nine strategies used in HD and ten in HH conditions, by the international players were respectively:

– Pre-cooling: cold drinks (22%, 33%), cold shower (18%, 27%), cold water spray (9%, 9%), cold packs (4%, 11%), cold water immersion (4%, 7%), cold towel (0%, 7%), menthol gel (0%, 7%), cold vest (0%, 4%), menthol ingestion (0%, 4%), ice slurry ingestion (0%, 2%).
– Per-cooling: cold towel (47%, 53%), cold drinks (38%, 42%), cold packs (31%, 24%), cold water spray (20%, 18%), ice slurry ingestion (0%, 4%,) menthol gel (2%, 0%), cold vest (0%, 2%).
– Post-cooling: cold shower (71%, 73%), cold drinks (38%, 42%), cold water immersion (31%, 42%), cold packs (16%, 18%), cold water spray (7%, 7%), cold towel (7%, 11%), menthol gel (2%, 7%), cold vest (2%, 2%), menthol ingestion (2%, 2%), ice slurry ingestion (0%, 2%).

The differences in the use of cooling strategies by environments and players’ levels, which were previously detected, mostly observed during the per-cooling phase as evidenced by the strong correlation of *χ*^2^ (*R*^2^ = 0.82), compared to the pre- and post-cooling phases (*R*^2^ = 0.38 and 0.32, respectively). For all statistics, please see the Supplementary results.

### 3.5 Justification

Regarding the justification of cooling strategies employed by regional players in HD and HH respectively, they reported: personal research (34%, 27%), tested and found to be effective (26%, 27%), available on site (20%, 25%), proposed by staff/trainer (13%, 11%), no justification (7%, 7%).

National players justified the use of strategies in HD and HH conditions by: tested and found to be effective (35%, 31%), personal research (29%, 25%), available on site (18%, 22%), proposed by staff/trainer (8%, 11%), no justification (10%, 10%).

The justification of the international players, regarding cooling strategies employed in HD and HH conditions, respectively, were: tested and found to be effective (26%, 25%), personal research (22%, 28%), proposed by staff/trainer (28%, 26%), available on site (21%, 20%), no justification (2%, 1%).

### 3.6 Perceived effectiveness

Regional athletes perceived effectiveness of cooling strategies, in HD and HH conditions, respectively, as being:

– Between 4 (“*Effective for minimising performance impairments*”) and 3 (“*Sometimes effective and sometimes not effective*”) for: cold water immersion (M = 3.8, M = 3.7), cold shower (M = 3.7, M = 3.7), cold packs (M = 3.7, M = 3.8), cold drinks (M = 3.4, M = 3.4)
– Between 3 (“*Sometimes effective and sometimes not effective*”) and 2 (“*Not effective for minimising performance impairments*”) for: cold towel (M = 2.5, M = 2.5)
– Equal to 2 (“*Not effective for minimising performance impairments*”) for ice slurry ingestion (M = 2, M = 2) and cold water spray (M = 2, M = 2).

Regarding national athletes, perceived effectiveness of cooling strategies, in HD and HH conditions, respectively, were:

– Between 4 and 3 for: cold towel (M = 4, M = 3.5), cold packs (M = 3.9, M = 3.6), cold shower (M = 3.9, M = 3.8), cold water immersion (M = 3.7, M = 3.5), menthol ingestion (M = 3.5, M = 3), cold drinks (M = 3.2, M = 3.3).
– Between 3 and 2 for: cold water spray (M = 3.0, M = 3.0), ice slurry ingestion (M = 2, M = 2.5), and menthol gel (M = 0, M = 2.6).

International athletes rated perceived effectiveness of cooling strategies, in HD and HH conditions, respectively, as:

– Between 4 and 3 for: cold vest (M = 3.8, M = 3.8), cold packs (M = 3.8, M = 3.7), cold shower (M = 3.7, M = 3.8), cold drinks (M = 3.8, M = 3.7), cold water immersion (M = 3.7, M = 3.7), cold water spray (M = 3.5, M = 3), menthol ingestion (M = 3.1, M = 3.2).
– Between 3 and 2 for: menthol gel (M = 3, M = 2.8), ice slurry ingestion (M = 2.5, M = 2.5), cold towel (M = 2.5, M = 2.2).

## 4 Discussion

This study aimed to investigate the pre-, per- and post-cooling strategies used by a sample of competitive tennis players of various levels of play who occasionally train and compete in HD and HH conditions.

Firstly, the results revealed that most athletes perceived a difference when playing in HD compared to HH conditions, with the hot and humid condition considered to be the most stressful. This result is consistent with previous studies that indicated that the physiological constraints amplified in HH compared to HD (10, 12, 13) are accompanied by psychological constraints such as increase in ratings of perceived exertion, thermal sensation, thermal discomfort and fatigue (9, 11).

The results of this study also revealed that tennis players experienced more symptoms related to heat-related illness when competing in HH than in HD. Indeed, national and international players reported more cramping, and more heat exhaustion for international players only, in HH than in HD. In the heat, the increase in sweating caused by physiological thermoregulatory processes (9) can induce, in tennis players, bodily dehydration and losses of electrolytes such as sodium and chloride (30–32), which can promote the development of heat cramps linked to high amounts of body fluid and sodium lost (7). Indeed, body weight losses [e.g., 5–6 liters of body fluid lost likely to be recorded at the end of game in international players (3)], have been generally reported during tennis matches played in HD conditions (31, 33). In addition, it is important to note that in HH, losses of body fluids and electrolytes are even greater than in HD (10). This is why, to prevent the appearance of cramps, it will therefore be important that tennis players start the competition being euhydrated and resort to regular and sufficient ingestion of exercise drinks containing sodium chloride when playing in the heat for more than one hour (19). In addition, good management hydration during exercise as well as regular breaks in the shade will help limit the onset of heat exhaustion (34), and could also prevent the occurrence of heat stroke, particularly if combined with cooling techniques (35).

Regarding type of cooling strategy, international players more frequently ingested ice slurry in HH than in HD. They also used more frequently cold water immersion and cold vest than regional and national players in HH; and cold packs and cold towel than the latter in both environmental conditions. The use of specific cooling strategies such as ice-slurry ingestion or cold vest, only by international players, could be explained by the non-availability of these among regional and national athletes. Indeed, our results revealed that regional and national players used more frequently cold drinks, which can be found everywhere, than international players. However, it is recommend that coaches and tournament organizers offer athletes different internal and external cooling strategies (3, 9, 36), and more particularly ice slurry and cold vest, which have recently been shown to be effective strategies for mitigating the development of thermal stress during outdoor tennis matches (24). If ice slurry and cold vest are not available, ingesting cold drinks before and during match can reduce body temperature (37, 38). It has been suggested that the addition of external cooling strategy (e.g., cold towel, cold packs, cold water spray) may result in greater reduction in thermal discomfort (39), but the choice of the strategies will depend on their availability, timing or the possibility of regulatory use in pre- (e.g., cold water immersion, water spray, cold vest or cold packs), per- (e.g., cold packs, cold vest, water spray or menthol cooling) or post-cooling (e.g., cold water immersion) during training or competition (40).

Whatever the level of expertise, it was observed that tennis players mainly employed cold drinks and cold shower as pre-cooling strategies. Except for cold packs, more frequently used by international players in HH, there was no other significant difference regarding the strategies employed. Previous studies (e.g., (40, 41) indicated that pre-cooling such as cold water ingestion could improve intermittent sprint exercise and endurance performance, in the heat. In addition, Jones et al. (42) revealed that cold water immersion may be the most effective pre-cooling strategy to improve endurance performance in the heat. However, the practical aspect must be taken into account, and the use of a cold shower, when a cold water immersion is not possible, can be considered a good alternative because it cools the whole body as recommended by Bongers et al. (40) and can lower baseline temperature (38) before exercising.

Differences in the use of cooling strategies were mostly observed during per-cooling. Regional and national players used more frequently cold drinks, than international players, in HD condition. In hot environments, regional players preferred using cold water spray whereas international athletes more frequently used cold towel in HH and cold packs in HD. Finally, menthol was more frequently used by national players, in both hot conditions. Research has shown that multiple per-cooling strategies can be used to tackle the decline intermittent exercise performance in HD and HH conditions (19). For example, Lynch et al. (25) showed beneficial effects of cold towel in reducing thermal strain, perceived exertion, and thermal sensation during tennis match-play in HD condition. The authors evoked that cold drink ingestion alone does not provide optimal cooling. Similarly, Naito et al. (43) found that cold water ingestion, during breaks in an exercise simulating tennis matches, was less effective than ice slurry in suppressing the rise in central temperature. Finally, a recent study showed that the combination of ice slurry ingestion and cold vest was more effective than each strategy used alone, in mitigating heat stress during outdoor tennis matches in HD condition (23), which leads us to suggest that athletes test not only one cooling strategy, but also combinations between different strategies to evaluate which would suit them best. Indeed, national and international players revealed that the justification regarding the use of cooling strategies was mainly based on the “test/found to be effective” item (secondly for the regional players). It therefore seems important that players can know that cooling strategies decrease the rate at which core temperature increases after exercise initiation (37) and perceived exertion, and can also improve thermal comfort and performances when exercising in the heat (21).

Finally, all players mainly employ as post-cooling strategies cold shower followed by cold drinks ingestion. International players more frequently used cold towel, cold packs and cold water immersion than regional players in both hot conditions. Post-cooling is used to reduce after match body and muscle temperatures, to reduce muscle soreness or perceived exertion after 24 h, and to improve recovery (40). These authors added that cold water immersion is particularly effective in reducing subjective symptoms. In addition, post-exercise cryotherapy, which none of the players in the current study reported using, could be beneficial in dampening the inflammatory response. It is also necessary to rehydrate through both fluids and foods, including amino-acids, carbohydrate, and electrolytes, after match-play tennis in HD and HH conditions, with an amount slightly above the body mass loss (22).

To benefit from information on cooling strategies that is more valid and complete than that obtained with personal research it seems necessary to offer seminars to the players and to deepen the formation of coaches/staff.

The main limitation of this study concerns the use of a self-report questionnaire. It is possible that despite definitions of variables such as heat-related illnesses, the number of some of them including heat stroke, has been overestimated, particularly for regional level players.

## 5 Conclusion

The aim of this study was to evaluate the type, justification and perceived effectiveness of the cooling strategies employed by competitive regional, national, and international tennis players in HD and HH environments. The results revealed that the athletes considered the HH to be more stressful than HD and experienced more heat-related illness in HH environments. It was found that tennis players used both internal (cold drinks) and external (cold shower, cold water spray, cold towel or cold water immersion) cooling strategies to combat the negative effects of environmental stress in HD and HH conditions. International players used cold packs and cold towel more frequently than the regional and national players in both hot conditions, and used cold water immersion and cold vest more frequently than the latter in HH environment. Depending on the timing and regardless level and environmental conditions, players mainly used cold drinks and cold shower as pre-cooling. Differences in strategy use were mostly observed during per-cooling where regional and national players more frequently used cold drinks in HD than international players who more frequently used cold towel in HH and cold packs in HD environments than the latter. Moreover, international more frequently used cold towel, cold packs and cold water immersion as post-cooling strategies than regional players. Finally, regardless of their level of expertise, players lack information regarding cooling and education for them and coaches is needed to improve the use of cooling strategies in hot environments.

## Data Availability

The raw data supporting the conclusions of this article will be made available by the authors, without undue reservation.
