# Supplementary material for: Pre-, Per- and post-cooling strategies used by competitive tennis players in hot dry and hot humid conditions
Source: Front Sports Act Living. 2024 Sep 18;6:1427066. doi: 10.3389/fspor.2024.1427066 (PMC11445033; doi:10.3389/fspor.2024.1427066)
Supplement: Supplementary file 2 [file Datasheet2.docx]

**Interviews**

To support the answers to the questionnaire, 3 athletes (1 from each level category: regional, national and international) also completed individual online-recorded interview to a better understanding of the player’s experiences concerning the use of cooling strategies (see Bayne et al., 2022 for similar procedure; and supplemental material file for the details). The interviews, considered as single case studies, were conducted in accordance with Keegan et al. (2017) guidelines via Zoom® cloud meeting. They were semi-structured and followed a discussion format based on location of training or competition performed, symptoms related to heat-related illness, and on questionnaire responses regarding timing, type, justification and effectiveness of their cooling strategies in HD as well as HH locations.

As indicated in the data analysis part, the recorded interviews were anonymized, transcribed and analysed according to the procedure of Braun and Clarke (2006). First, the first and last authors familiarized themselves with the data by reading the transcripts at least 3 times. They then individually generated initial codes before meeting to determine themes. Then, the investigators separately identified common themes and meanings in each interview, and jointly determined the themes. Next, the authors identified common patterns in the interview and organized them into themes and sub-themes to connect the experiences of the 3 tennis players. Moreover, the themes and sub-themes were defined and named separately and then collectively. Finally, the report was produced.

Regarding the **condition**, the regional athlete indicated: “*I have the impression that it is hotter, heavier when I play in HH*”. In addition, international athlete said: “*During HH tournaments, I feel more discomfort. I feel like I'm sweating a lot more: which isn’t pleasant”.*

For example, *c*oncerning the **symptom**, the national player reported: “*In Martinique (HH), my match was at 11 am. (…) It was so hot that at one point I was even on the verge of feeling unwell”.* Moreover, international player stated: “*I remember having cramps in Miami (HH) and being tired more quickly in this humid heat, whereas in Tunisia (HD), things were better*”.

For the **type of cooling strategies**, the regional athlete indicated: “*I used cold drinks when I play in the heat (…). In Guadeloupe (HH), I stayed at least 10 minutes in the shower”.*

The national player said: “*This summer (HD), I regularly drank cold water to limit the impact of the heat (…) At the Reunion (HH) I got into the habit of using cold towel when switching sides, I returned to the game feeling fresher and more concentrated*”.

The international athlete commented: “*Once in Tampa (HH), I tried cold vest. I liked it, I put it on during the warm-up and I had a great start to the match (…) Otherwise I use ice packs.”*

The international athlete commented: “*Once in Tampa (HH), I tried cold vest. I liked it, I put it on during the warm-up and I had a great start to the match (…) Otherwise I use ice packs.”*

Concerning **timing**, the regional athlete revealed: ”*Before and even during the match, my favorite strategy is: cold drinks. (…) On the other hand, what a joy to take a cold shower after playing, and if I could I would take a bath in the sea or the river. (…) Ice slurry ingestion? No, I've never tried it, but I'd rather see it after the effort because I'd be afraid of having my brain frozen if I took it before or during the match*.”

During interview, national player said: “*My coach talked to me a little about cooling strategies. So, during match, I use the cold water spray, but I don't like drinking cold water when I play. And afterwards, we saw together that showers or baths favoured recovery; (…) and it's true that it worked in Martinique (HH) where I had a series of singles and doubles matches and I had recovered better than last year when I hadn’t done anything except stretching.*”

The international player indicated: “*For 2 years, as soon as I play in the heat, before each match I take a shower and I drink cold water. (…) During the match, I put ice in the towel and put it on my neck at each switching side and if I haven't found ice I use ice packs (…) and after the matches I quickly take a cold shower. (…) I feel like I drink more in HH than in HD but I don't do anything different*”.

Regarding **justification***, t*he regional player said: “*I had to play a tournament in the south of France, and knowing that it was going to be very hot, I consulted discussion forums on the internet to see if there were any cooling techniques used by other players*”.

The national player indicated: “*I went to the ITF tournament in Saint-François (HH), there was a stand on cooling, and I spoke with experts. They advised me to test the strategists in training before using them in matches, which I did when I returned to the academy*”.

The international player commented: “*We established the cooling techniques that could be beneficial, and when to apply them (before, during or after matches) with our staff. (…) I tested different cooling strategies and now I have my personal program that I have to follow when playing in HD or HH (…) I use the same program in both climates. I would have liked to use crushed ice or cold packs more often, but the problem is availability at the hotel or tournament.”*

For **perceived effectiveness**, a regional player indicated “*Personally, I try to bring a bottle of cold water when it's hot, and I especially like the shower after exercise (…) I feel like this is what works best for me (…) I tried the cold towel: at first it was good but after the ice melted and it had no effect. (…) It's true that I didn't have a cooler to keep it cool”.*

A national athlete declared: “*When I manage to get ice at the hotel, I put it in a plastic bag and wrap it in my towel to use during switching sides, it's effective. (…) And to recover, there's nothing better than a good cold shower or a cold bath*. *And sometimes I allow myself a cold beer (laughs)”.*

A international player said: “*I have integrated the use of cold drinks (water and exercise drink) as well as ice packs into my switching side routine; it is effective for cooling down. (…) Before starting the match, if I see that it is going to be very hot, I take a shower: I think it’s great because I feel fresh for the first games*. (…) *I also tried the menthol gel, the sensation was pleasant during the match but I find myself using it more after matches.*”
